# Research on the Longitudinal Section of River Restoration Using Probabilistic Theory

**DOI:** 10.3390/e23080965

**Published:** 2021-07-27

**Authors:** Yeon-Moon Choo, Ji-Min Kim, Ik-Tae An

**Affiliations:** Department of Civil and Environmental Engineering, Pusan National University, Busan 46241, Korea; chooyean@naver.com (Y.-M.C.); giveme2077@korea.kr (J.-M.K.)

**Keywords:** informational entropy, mean river slope, river slope, nonlinear regression analysis, longitudinal elevation

## Abstract

Since the 1960s, many rivers have been destroyed as a consequence of the process of rapid urbanization. As accurate figures are important to repair rivers, there have been many research reports on methods to obtain the exact river slope and elevation. Until now, many research efforts have analyzed the river using measured river topographic factors, but when the flow velocity changes rapidly, such as during a flood, surveying is not easy; and due to cost, frequent measurements are difficult. Previous research has focused on the cross section of the river, so the information on the river longitudinal profile is insufficient. In this research, using informational entropy theory, equations are presented that can calculate the average river slope, river slope, and river longitudinal elevation for a river basin in real time. The applicability was analyzed through a comparison with the measured data of river characteristic factors obtained from the river plan. The parameters were calculated using informational entropy theory and nonlinear regression analysis using actual data, and then the longitudinal elevation entropy equation for each river and the average river slope were calculated. As a result of analyzing the applicability of the equations presented in this study by R^2^ and Root Mean Square Error, all R^2^ values were over 0.80, while RMSE values were analyzed to be between 0.54 and 2.79. Valid results can be obtained by calculating river characteristic factors.

## 1. Introduction

While there has been a lot of research on the cross-section of rivers, there is not enough information on the longitudinal section, as there has not been much research on it. The longitudinal elevation and river slope are difficult to measure directly, especially when the flow velocity changes rapidly. However, owing to the development of measurement technology, it is now easy to obtain data such as river slopes and river longitudinal elevations through Light Detection and Ranging (LIDAR), which makes it easier to produce a Digital Elevation Model. However, using the data obtained through LIDAR, there are indisputable errors between the digital elevation models and measured values [1]. To resolve this problem, entropy theory was applied as a means of obtaining topographic factor data while minimizing errors.

## 2. Methodology

In recent years, the concepts of statistics, entropy, and probability have been often used to analyze rivers, and the method of calculating river Longitudinal Elevation studied in this paper can also be induced by maximizing entropy.

Lienhard derived the dimensionless unit water level of the drainage basin in a statistical method using the concept of the maximum entropy expression [2]. The concept of entropy was studied as a scientific discipline by Wilson [3], while Leopold and Langbein applied the concept of entropy to study river behavior [4]. Yang derived the river mean elevation law using the concept of physical entropy [5]. Singh and Fiorentino broadened the scope of the concept of information entropy [6]. Meanwhile, Chapman [7] used the concept of information entropy for the quantitative analysis of the uncertainty of hydrologic data.

Chiu introduced the concept of information entropy to calculate a two-dimensional flow velocity formula and calculated a flow velocity probability density function that satisfied the constraints on the maximum entropy law [8,9,10]. Fiorentino et al. defined the river longitudinal section by identifying the relationship between information entropy and the basin average elevation, and compared the river longitudinal section with actual measurements [11,12].

Recently, Mirauda et al. [13] and Kundu [14] analyzed the location of the dip phenomenon occurring in an open channel using entropy models. Although the concept of entropy has been widely studied as a scientific discipline, not many cases have been used to study river longitudinal sections.

In this study, formulae for calculating the river slopes, average river slopes, and river longitudinal elevation are suggested using concepts similar to those of Chiu. Maximizing entropy theory can be used to determine the parameters and to obtain the river longitudinal elevation before destruction. This can be used to make the river restoration model.

## 3. Theoretical Background

### 3.1. Nonlinear Regression

In statistics, regression analysis is used to model correlations between dependent and independent variables. Nonlinear regression models regress expressions using nonlinear prediction functions, and unknown parameters are estimated from the equation, which are called regression models. In general, for regression analysis, the basic method is to obtain parameters that minimize the sum of squares of residuals. For nonlinear regression, it is impossible to mathematically represent the error, and this can only be obtained by iterative methods. The regression model is conducted using the least squares method. If the purpose of the regression model is prediction rather than interpretation, nonlinear regression is used. This is because nonlinear models can also be modeled on data with complex patterns. Therefore, nonlinear regression analysis is used in this paper to predict the river longitudinal section by calculating river slopes and river longitudinal altitudes.

### 3.2. Entropy Theory

Since natural phenomena proceed in the direction of increasing entropy, the entropy equation can explain natural phenomena. From a mathematical point of view, river slopes and river altitudes can also be expressed using maximum entropy laws. There are three types of entropy that can quantify the characteristics of the system and be used to build a model of the system: thermodynamic entropy, statistical entropy, and information entropy. The probability distribution function of a mathematical variable can be determined by maximizing the entropy of the variable in a particular way, and from a physical point of view the maximized entropy of a river longitudinal section creates a constant probability distribution under constraints. Thus, the probability law of arbitrary river longitudinal sections and the corresponding entropy generally depends on constraints, and the most important point of an entropy-based approach is to determine how to constrain a situation. The probability density function p(x) for continuous state variables x is quantitatively represented by entropy, such as in Equation (1):

(1) $$H=-\int_{-\infty }^{\infty }p\left(x\right)lnp\left(x\right)dx$$

Entropy H, defined as Equation (1), represents the uncertainty or randomness of the state variable x, and p(x)∙dx represents the probability between the state variable x and the state variable x+dx. Equation (2) is defined as the mean of a continuous random variable $\bar{x}$:

(2) $$\bar{x}=\int_{-\infty }^{\infty }xp\left(x\right)dx$$

(3) $$\int_{-\infty }^{\infty }p\left(x\right)dx=1$$

**Figure 1:**
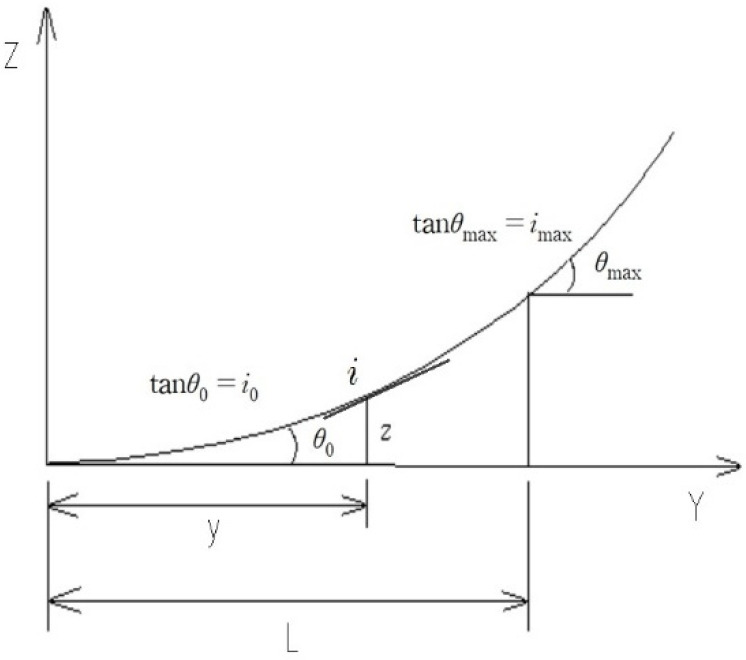
River longitudinal section elevation (starting from z = 0).

Figure 1 shows the longitudinal section of a stream, which is the altitude z at any elevation from its initial point, y is the horizontal distance, and the slope at that point is i. The slope of the initial point is $\tan\theta_{0}=i_{0}$, and the slope of the end point is $\tan\theta_{max}=i_{max}$. If a river basin is considered a system in which i is a river slope, and the state variable x of the system is defined in Equation (1), the entropy of the basin can depend on the probability. The probability density function of the basin is the same as Equation (4):

(4) $$H=-\int_{i_{0}}^{i_{max}}p\left(i\right)lnp\left(i\right)di$$

where p(i) is probability density function for slope i.

The first constraint is Equation (5), expressed using river slopes instead of state variations in Equation (2), and the second constraint is Equation (6), using the definition of general probability. By maximizing entropy, the probability density function p(i) for river slopes i can be obtained. The p(i)∙di is the probability of a state variable I and is expressed as Equation (6):

(5) $$\bar{i}=\int_{i_{0}}^{i_{max}}ip\left(i\right)di$$

(6) $$\int_{i_{0}}^{i_{max}}p\left(i\right)di=1$$

Arranging the independent constraint conditions can be given as Equation (7):

(7) $$\int_{a}^{b}\Phi_{j}\left(i,p\right)di\;\;j=1,\;2$$

where a is the minimum value of i, b is the maximum value of i, and j is the constraint number (j=1 is Equation (5), j=2 is Equation (6)).

Therefore, $p\left(i\right)$, which maximizes the entropy, can be obtained using the method by Lagrange as Equations (8)–(10):

(8) $$\frac{\partial I\left(i,p\right)}{\partial p}+\overset{2}{\underset{j=1}{\sum }}\lambda_{j}\frac{\partial \phi_{j}\left(i,p\right)}{\partial p}=0$$

(9) $$I\left(i,p\right)=-p\left(i\right)lnp\left(i\right)$$

where $\phi_{1}\left(i,p\right)=p\left(i\right),\;\phi_{2}\left(i,p\right)=i\cdot p\left(i\right)$.

(10) $$\frac{\partial \phi_{1}\left(i,p\right)}{\partial p}=1,\;\frac{\partial \phi_{2}\left(i,p\right)}{\partial p}=i$$

where $\lambda_{1}$ and $\lambda_{2}$ are Lagrange multipliers.

Substituting Equations (12) and (13) into Equation (11) can be constructed as the following Equation (14):

(11) $$-1-lnp\left(i\right)+\lambda_{1}+\lambda_{2}i=0$$

where $\lambda_{1}-1=b_{1},\;\lambda_{2}=b_{2}$ are the Lagrange multipliers.

Differentiating Equation (11) by p(i), Equation (12) shows the probability density function p(i).

Here, λ_1_ − 1 is $b_{1}$, and λ is $b_{2}$.

The organized formula is as follows:

(12) $$p\left(i\right)=e^{b_{1}+b_{2}i}$$

Then, b_1_ is obtained by substituting the probability density function p(i) expressed in Equation (12) into the constraint Equation (6). The result is Equation (13), and the Lagrange multiplier b_1_ is Equation (14):

(13) $$\int_{i_{0}}^{i_{max}}p\left(i\right)di=\int_{0}^{i_{max}}p\left(i\right)di-\int_{0}^{i_{0}}p\left(i\right)di=\frac{e^{b_{1}}}{b_{2}}\left(e^{b_{2}\cdot i_{max}}-e^{b_{2}\cdot i_{0}}\right)=1$$

(14) $$e^{b_{1}}=\frac{b_{2}}{e^{b_{2}i_{max}}-e^{b_{2}i_{0}}}$$

The variable b_1_ of the probability density function Equation (12) is eliminated by substituting b_1_ into Equation (14), and the probability density function p(i) follows as Equation (15):

(15) $$p\left(i\right)=e^{b_{1}+b_{2}\cdot i}=\frac{b_{2}}{e^{M_{max}}-e^{M_{0}}}\cdot e^{b_{2}\cdot i}\;\;$$

### 3.3. River Mean Slope Formula Development

The probability density function p(i) is applied to the river mean slope $\bar{i}$ of Equation (5). Equation (16) is integrated, and M_max_ is substituted for $b\cdot i_{\max}$., and M_0_ for $b\cdot i_{0}$. The result is the same as Equation (17):

(16) $$\bar{i}=\int_{i_{0}}^{i_{max}}ie^{b_{1}+b_{2}i}di=e^{b_{1}}\int_{i_{0}}^{i_{max}}ie^{b_{2}i}di$$

(17) $$\int_{i_{0}}^{i_{max}}ie^{b_{2}i}di=\int_{0}^{i_{max}}ie^{b_{2}i}di-\int_{0}^{i_{0}}ie^{b_{2}i}di=\frac{1}{b_{2}}\left(M_{max}e^{M_{max}}-M_{0}e^{M_{0}}-e^{M_{max}}+e^{M_{0}}\right)\;={\left(\frac{i_{max}+i_{0}}{M_{max}+M_{0}}\right)}^{2}\times \left(M_{max}e^{M_{max}}-M_{0}e^{M_{0}}-e^{M_{max}}+e^{M_{0}}\right)$$

The river mean slope $\bar{i}$ is expressed in Equations (18) and (19):

(18) $$\bar{i}=\frac{e^{b_{1}}}{b_{2}{}^{2}}\left(M_{max}e^{M_{max}}-M_{0}e^{M_{0}}-e^{M_{max}}+e^{M_{0}}\right)$$

(19) $$\bar{i}=\left(\frac{i_{max}+i_{0}}{M_{max}+M_{0}}\right)\times \frac{\left(M_{max}e^{M_{max}}-M_{0}e^{M_{0}}-e^{M_{max}}+e^{M_{0}}\right)}{\left(e^{M_{max}}-e^{M_{0}}\right)}$$

The river mean slope i is determined by the slope of the initial and final points, and the entropy parameter $M_{0},\;M_{max}$.

(20) $$\int e^{b_{2}\cdot i}di=\frac{1}{L}\cdot \frac{1}{e^{b_{1}}}\int dy$$

Integrating Equation (20) yields Equation (21), and the river slope is expressed in Equation (22):

(21) $$e^{b_{2}\cdot i}=\frac{b_{2}}{L\cdot e^{b_{1}}}\cdot y+e^{M_{0}}$$

(22) $$i=\frac{1}{b_{2}}\ln\left[\frac{y}{L}\cdot \frac{b_{2}}{e^{b_{1}}}+e^{M_{0}}\right]$$

Then, b_1_, b_2_ are eliminated by substituting b_1_ of Equation (14) into Equation (22), and the result follows as Equation (23):

(23) $$i=\left(\frac{i_{max}+i_{0}}{M_{max}+M_{0}}\right)\ln\left[\frac{y}{L}\left(e^{M_{max}}-e^{M_{0}}\right)+e^{M_{0}}\right]$$

The river slope of a random point i is defined as dz/dy, and dz follows as Equation (24):

(24) $$\mathrm{dz}=\left(\frac{i_{max}+i_{0}}{M_{max}+M_{0}}\right)\times \ln\left[\frac{y}{L}\left(e^{M_{max}}-e^{M_{0}}\right)+e^{M_{0}}\right]\mathrm{dy}$$

By integrating Equation (24), the river elevation z can be obtained as Equation (27):

(25) $$z=\left(\frac{i_{max}+i_{0}}{M_{max}+M_{0}}\right)\left(\frac{L}{e^{M_{max}}-e^{M_{0}}}\right)\;\times \left[\left\{e^{M_{0}}+\left(e^{M_{max}}-e^{M_{0}}\right)\frac{y}{L}\right\}\times ln\left\{e^{M_{0}}+\left(e^{M_{max}}-e^{M_{0}}\right)\frac{y}{L}\right\}-\left\{+\left(e^{M_{max}}-e^{M_{0}}\right)\frac{y}{L}\right\}+c\right]$$

Here, c is an integral constant, so if the horizontal distance is y = 0, then the river longitudinal elevation is z = 0. Therefore, the integral constant is $c=e^{M_{max}}-M_{0}e^{M_{0}}$. Substituting c into Equation (25), the river elevation z can be shown as Equation (26).

We defined M_max_ as the product of b and i_max_, and M_0_ as the product of b and i_0_. Thus, Equation (25) could be written as Equations (26) and (27):

(26) $$z=\left(\frac{i_{max}-i_{0}}{M_{max}-M_{0}}\right)\left(\frac{L}{e^{M_{max}}-e^{M_{0}}}\right)\;\times \left[\left\{e^{M_{0}}+\left(e^{M_{max}}-e^{M_{0}}\right)\frac{y}{L}\right\}\times ln\left\{e^{M_{0}}+\left(e^{M_{max}}-e^{M_{0}}\right)\frac{y}{L}\right\}-\left\{+\left(e^{M_{max}}-e^{M_{0}}\right)\frac{y}{L}\right\}+e^{M_{max}}-M_{0}e^{M_{0}}\right]$$

(27) $$z=\frac{1}{b}\left(\frac{L}{e^{M_{max}}-e^{M_{0}}}\right)\;\times \left[\left\{e^{M_{0}}+\left(e^{M_{max}}-e^{M_{0}}\right)\frac{y}{L}\right\}\times ln\left\{e^{M_{0}}+\left(e^{M_{max}}-e^{M_{0}}\right)\frac{y}{L}\right\}-\left\{+\left(e^{M_{max}}-e^{M_{0}}\right)\frac{y}{L}\right\}+e^{M_{max}}-M_{0}e^{M_{0}}\right]$$

### 3.4. RMSE

The Root Mean Square Error, RMSE, is a measure of the residual, which is the difference between the values predicted by the model and the actual observed values. The RMSE enables the predictive power to be integrated into a single unit of measurement. The RMSE of the model’s prediction for the estimated variable $X_{est,i}$ is defined as the square root of the mean square error (Equation (28)):

(28) $$\mathrm{RMSE}=\sqrt{\frac{\sum_{i=1}^{n}{\left(X_{obs,i}-X_{est,i}\right)}^{2}}{n}}$$

where $X_{obs,i}$ indicates the actual observed value, and $X_{est,i}$ is the predicted value obtained from the model.

## 4. Application to Real River

This study compared and analyzed the measured and theoretical values obtained by the river mean slope formula. The river longitudinal elevation was obtained by the River Improvement Plan, which is considered reliable, and the initial slopes, last slopes, and lengths of the seven rivers are shown in Table 1. The length of Nakdong river is 510 km, but the data used in this research was only partial due to the long length. The river slope used in this research was used by all positive river slopes.

### 4.1. Determination of Parameter by Measured Values

Based on the river longitudinal section, the parameters were yielded by a nonlinear regression analysis of the river longitudinal value z in order to make the error sum of the predicted values as small as possible. The Equations (26) and (27) were used for the calculation. Table 2 shows the parameter value, while Table 3 compares the average slope of the river calculated by substituting the parameters of each river.

### 4.2. River Longitudinal Elevation

**Figure 2:**
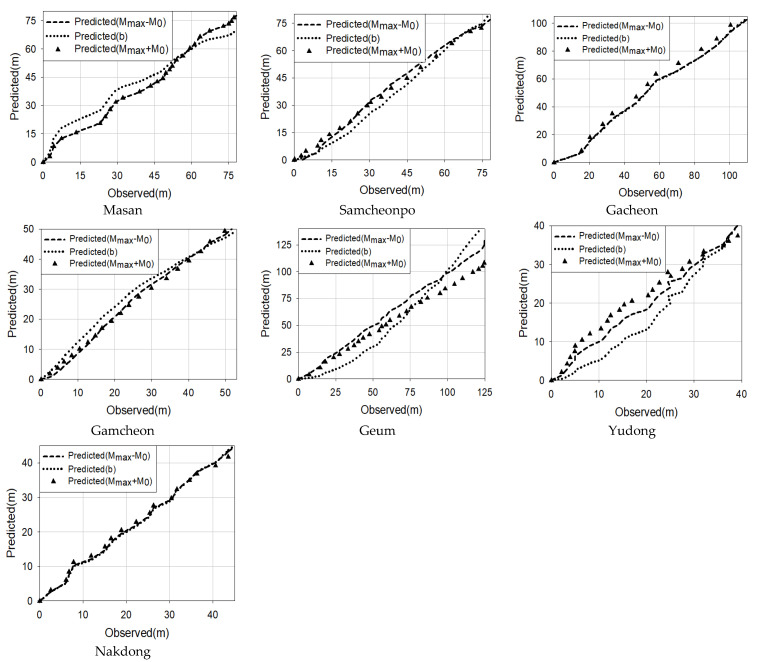
River longitudinal elevation.

The parameters of the topological factors can be obtained through the measurement of the elevation of the river and the nonlinear regression of the suggested formula, respectively. The results are compared to the actual measurement of the river’s longitudinal elevation calculated using the equation, and the accuracy of the prediction is evaluated using R^2^. Figure 2 shows the comparison for each method. Table 4 shows the accuracy analysis comparison. Considering that the values of R^2^ were all above 0.80, the formula suggested in this paper was meaningful in calculating the river elevation and slope.

## 5. Conclusions

To date, many studies have analyzed rivers based on survey results, but there is the disadvantage that in the event of disasters, such as floods, in situations where measurement is impossible, many data cannot be obtained.

To resolve this, this study suggests obtaining the prediction formula of the river longitudinal section using information entropy theory. In addition, while existing studies have studied the river elevation with one parameter, in this study two parameters have been considered in order to calculate river characteristic factors more accurately than in conventional studies. In this study, information entropy theory was used to calculate the average slope, river slope, and river longitudinal elevation. Using the suggested river longitudinal elevation equation, parameters representing river characteristics were determined based on the average slope and river longitudinal section in the basin.

(1) The verification of the accuracy of the equation in this paper is based on the nonlinear regression analysis with SPSS 26 and SYSTAT 6.0. The value is 0.8150 to 0.9950. The values show that the equation is valid, and the application to actual rivers is considered significant.
(2) Gacheon river shows the highest accuracy of prediction (close to 0.99) out of all methods. Yoodong river has parts that do not increase monotonically, and all three methods predict that singularity similarly. The suggested formulae are able to predict the section where the slope changes are large.
(3) Since M_max_ and M_0_ are parameters of the river, once the parameter is calculated the longitudinal section of the river can be obtained before it is destroyed. The reliability of this method can be further enhanced by using the data measured over 40 years or over 100 years.
(4) When making calculations using the equation presented in this study, it is easy to calculate the slope and elevation at a random point in the river basin.
(5) It is expected that one use the river longitudinal section obtained through the equation to restore damaged rivers to their longitudinal elevations and ramps before development.
(6) Using the method proposed in this paper, the river elevation can be obtained more accurately, which can help more precisely in the production of a digital elevation model or modify the data in places where it is hard to measure.

## Figures and Tables

**Table 1:** River slopes and lengths.

River	Length (km)	$i_{0}$	$i_{max}$
Masan	18.57 (entire)	$1.1\times 10^{-1}$	$0.9\times 10^{-2}$
Samcheonpo	7 (entire)	$6.3\times 10^{-2}$	$1.1\times 10^{-1}$
Gacheon	21.19 (entire)	$1.3\times 10^{-2}$	$3.18\times 10^{-2}$
Gamcheon	13.13 (entire)	$1.27\times 10^{-2}$	$1.1\times 10^{-2}$
Geum	3.25 (entire)	$3.0\times 10^{-2}$	$3.81\times 10^{-2}$
Yudong	11.31 (entire)	$4.29\times 10^{-4}$	$5.25\times 10^{-3}$
Nakdong	21.42 (selected)	$3.2\times 10^{-2}$	$2.5\times 10^{-2}$

**Table 2:** Parameter by each method.

River	$M_{max}+M_{0}$		$M_{max}-M_{0}$		b
	$M_{0}$	$M_{max}$	$M_{0}$	$M_{max}$	
Masan	−44.778	−8.481	−53.336	−8.481	−104.898
Samcheonpo	−1311.532	−708.388	−2.056	3.428	−5.911
Gacheon	−48.3386	−21.8701	−21.2442	−47.2009	−602.821
Gamcheon	1	0.9944	−24.804	5.113	−88,000
Geum	−2.6198	−101.655	−2.6195	−13.8264	−456.546
Yudong	−15.179	−9.616	−2.517	−8.917	39.952
Nakdong	−155.335	−20.034	−11.929	−91.464	−2458.048

**Table 3:** Mean slope comparison between the measured and the predicted values.

River	Measured	Predicted		
		$M_{max}+M_{0}$	$M_{max}-M_{0}$	b
Masan	0.01818	0.022464	0.022648	0.019296
Samcheonpo	0.054015	0.05124	0.194054	0.054411
Gacheon	0.015634	0.015098	0.01483	0.014972
Gamcheon	0.001508	0.001588	0.001247	0.001284
Geum	0.001591	0.001052	0.000876	0.001145
Yudong	0.003771	0.002601	0.002624	0.002942
Nakdong	0.028358	0.003568	0.00355	0.003488

**Table 4:** Accuracy analysis results.

River	$M_{max}+M_{0}$		$M_{max}-M_{0}$		b	
	R^2^	RMSE	R^2^	RMSE	R^2^	RMSE
Masan	0.987	2.153	0.987	2.153	0.938	5.883
Samcheonpo	0.94	0.828	0.991	2.72	0.979	4.175
Gacheon	0.995	2.794	0.987	4.908	0.987	5.142
Gamcheon	0.998	0.549	0.992	1.321	0.966	2.862
Geum	0.998	10.821	0.955	1.605	0.815	15.775
Yoodong	0.995	2.687	0.949	0.822	0.94	3.831
Nakdong	0.993	1.369	0.995	0.834	0.994	0.801
